# Effective coverage of antenatal care services in Ethiopia: a population-based cross-sectional study

**DOI:** 10.1186/s12884-024-06536-6

**Published:** 2024-04-27

**Authors:** Zewditu Abdissa, Kassahun Alemu, Seblewengel Lemma, Della Berhanu, Atkure Defar, Theodros Getachew, Joanna Schellenberg, Tanya Marchant, Solomon Shiferaw, Amare Tariku, Tadesse Guadu, Girum Taye, Meseret Zelalem, Lars Ake Persson

**Affiliations:** 1 Institute of Public Health, College of Medicine and Health Sciences, University of Gondar, Gondar, Ethiopia; 2 Department of Anesthesia, School of Medicine, College of Medicine and Health Sciences, University of Gondar, Gondar, Ethiopia; 3 Department of Epidemiology and Biostatistics, Institute of Public Health, College of Medicine and Health Sciences, University of Gondar, Gondar, Ethiopia; 4 Department of Disease Control, Faculty of Infectious and Tropical Diseases, London School of Hygiene and Tropical Medicine, London, UK; 5 Health System and Reproductive Health Research Directorate, Ethiopian Public Health Institute, Addis Ababa, Ethiopia; 6 School of Public Health, Addis Ababa University, Addis Ababa, Ethiopia; 7 Department of Nutrition, Institute of Public Health, College of Medicine and Health Sciences, University of Gondar, Gondar, Ethiopia; 8 Department of Environmental Health, Institute of Public Health, College of Medicine and Health Sciences, University of Gondar, Gondar, Ethiopia; 9 Maternal, Child and Adolescent Health Service Lead Executive Office, Federal Ministry of Health, Addis Ababa, Ethiopia; 10 Department of Pediatrics and Child Health, School of Medicine, College of Medicine and Health Sciences, University of Gondar, Gondar, Ethiopia

**Keywords:** Effective coverage Input-adjusted coverage, Intervention coverage, Quality-adjusted coverage, Facility readiness, Process quality, Ethiopia

## Abstract

**Background:**

Antenatal care (ANC) is a principal component of safe motherhood and reproductive health strategies across the continuum of care. Although the coverage of antenatal care visits has increased in Ethiopia, there needs to be more evidence of effective coverage of antenatal care. The 'effective coverage' concept can pinpoint where action is required to improve high-quality coverage in Ethiopia. Effective coverage indicates a health system's performance by incorporating need, utilization, and quality into a single measurement. The concept includes the number of contacts, facility readiness, interventions received, and components of services received. This study aimed to measure effective antenatal care coverage in Ethiopia.

**Methods:**

A two-stage cluster sampling method was used and included 2714 women aged 15–49 years and 462 health facilities from six Ethiopian regions from October 2019 to January 2020. The effective coverage cascade was analyzed among the targeted women by computing the proportion who received four or more antenatal care visits where the necessary inputs were available, received iron-folate supplementation and two doses of tetanus vaccination according to process quality components of antenatal care services.

**Results:**

Of all women, 40% (95%CI; 38, 43) had four or more visits, ranging from 3% in Afar to 74% in Addis Ababa. The overall mean health facility readiness score of the facilities serving these women was 70%, the vaccination and iron-folate supplementation coverage was 26%, and the ANC process quality was 64%. As reported by women, the least score was given to the quality component of discussing birth preparedness and complication readiness with providers. In the effective coverage cascade, the input-adjusted, intervention-adjusted, and quality-adjusted antenatal coverage estimates were 28%, 18%, and 12%, respectively.

**Conclusion:**

The overall effective ANC coverage was low, primarily due to a considerable drop in the proportion of women who completed four or more ANC visits. Improving quality of services is crucial to increase ANC up take and completion of the recommended visits along with interventions increasing women’s awareness.

**Supplementary Information:**

The online version contains supplementary material available at 10.1186/s12884-024-06536-6.

## Background

Quality antenatal care (ANC) is one of the strategies to improve the health of the mother and her unborn child [1]. It provides an opportunity to identify complications early, offers timely management, and helps to build rapport with the mother about her health and the well-being of her unborn baby [2].

A previous study has reported that quality antenatal care increased the likelihood of using skilled birth attendance and post-natal care [3]. A review of studies from 28 African countries showed that quality ANC led to mothers’ utilization of subsequent maternal health services, such as skilled birth attendance, and consequently reduced maternal mortality. Previous studies from low- and middle-income countries have indicated that quality ANC decreased neonatal deaths by 34% [4, 5].

A mere visit to a healthcare facility by an expectant mother doesn't bring the desired effect unless care is provided by qualified professionals in environments where the required, infrastructure, essential interventions, and equipment are available, and all the essential ANC components are given as per the recommended process quality [6].

The World Health Organization recommended 49 interventions under five major areas as essential components of antenatal care [1]. These include nutrition, maternal and fetal assessment, preventive measures, interventions for common physiological symptoms, and interventions to improve quality antenatal care. Advice on healthy eating and daily iron- folate supplementation are among the nutrition components. Among other preventive interventions, providing two doses of tetanus vaccination is crucial to prevent neonatal tetanus. Low tetanus vaccination coverage increases the risk of neonatal tetanus and death in home deliveries with unhygienic conditions [7]. Similarly, inadequate coverage of iron-folate supplementation increases the risk of severe anemia and complications for mothers and newborns [8].

Effective coverage indicates a health system's performance by incorporating need, utilization, and quality into a single measurement [6]. It captures whether health services reach a target population with recommended service contacts and quality. Previous authors have used a variety of techniques to measure effective coverage. The combination of three factors, namely the target population, contact coverage, and quality of healthcare interventions, was a typical approach to measuring effective coverage [9]. Others also included the user adherence-adjusted and outcome adjusted-coverage in the effective coverage measurement [10].

Because of this variation in approaches, WHO and UNICEF organized the Effective Coverage Think Tank Group, which recommended that effective coverage be measured using a cascade approach with seven indicators: the population in need of the health service, the service contact coverage, the facility readiness, the coverage of the recommended health interventions, the quality-of-service provision, the extent of adherence of the user to the health service and the health outcome [11].

Women in several low-income countries have few ANC service contacts [10], and services provided are of low quality [13]. The distribution of ANC coverage and quality vary by socio-economic status and place of residence [12, 13].

In Ethiopia, although efforts were made to expand services to primary-level facilities, maternal healthcare service utilization remained low. According to the mini-EDHS 2019, when four or more ANC visits was in effect at the time of data collection, only 43% of the population completed this number of visits, 48% of births were attended by skilled attendants, and 13% of mothers received post-natal care within two days of delivery [14]. Ethiopia has been successful in reducing maternal mortality over the last decades. However, the neonatal mortality rate has remained stagnated, necessitating additional efforts [15].

Previous studies in Ethiopia assessing services provided to pregnant women mainly focused on ANC service coverage. Only a few studies addressed quality dimensions [16, 17], and one study measured content-adjusted coverage [18]. Therefore, this study aimed to determine effective ANC coverage in Ethiopia by taking into account the completion of the recommended ANC visits (contact coverage), the proportion who visited facilities where the necessary inputs were available (input-adjusted), the proportion who received the recommended interventions in the facilities with necessary inputs (intervention-adjusted), and the proportion who received the recommended interventions in the facilities with necessary inputs, as per the recommended process quality (quality-adjusted).

## Methods

### Study setting

This paper is based on a secondary analysis of data from the Performance Monitoring for Action Ethiopia (PMA) project [19]. PMA Ethiopia is implemented in collaboration between Addis Ababa University, Johns Hopkins University, and the Federal Ministry of Health to produce policy-relevant reproductive, maternal, neonatal, and child health results. Household and health facility data were collected from five regions and one city administration: Tigray, Afar, Amhara, Oromia, the Southern Nations, Nationalities and Peoples regions, and Addis Ababa. In Afar, only rural and in Addis Ababa, only urban strata were included, while the rest had both.

### Study design and data

This is a secondary analysis of data from a longitudinal study of pregnant and postpartum women aged 15–49 years from five regions and one city administration, at initial screening, six weeks, six months, and one year after birth. We analyzed the six-week postpartum interview along with the PMA cross-sectional data assessing facility readiness.

Data were collected from 2714 women aged 15–49 years and 462 health facilities from five regions and one city administration from October 2019 to January 2020.

Data on antenatal care visits collected from women assessed at initial screening and six weeks postpartum were linked to the facility data assessing maternal health service delivery points for the included women. We used an ecological linking method [20] to link facility readiness assessment data to women’s data using enumeration ID, as the same enumeration ID was given for the clusters from which households and health care facilities were selected.

### Eligibility

For this analysis, we included consenting pregnant or postpartum women 15–49 years old who were regular household members. The analysis also included data on healthcare facilities in the enumeration areas where the eligible women resided.

### Sampling and sample size

Household data were collected based on two-stage cluster sampling. Sample size was estimated to detect a 5% difference between groups of women defined by various reproductive, maternal and child health indicators. Considering an alpha level of 0.05 and a power of 0.8, a minimum sample size of 3100 women was needed [19].

### Data collection

Trained field workers collected data. The training included a review of survey protocols, questionnaire content, and interview skills. In addition to classroom exercises, field staff training included three days of field exercises, during which data collectors practiced using the tools on the data collection device (phones) [19]. The following background characteristics and service details were included in the study: region, residence, household wealth quintile, family size, age of the woman, marital status, gestational age at first ANC visit, number of ANC visits, service provider at first ANC visit, components of ANC received, any complication during recent pregnancy, and partner's encouragement to utilize maternal health services. Interviews were performed at around seven weeks, (median 7.4, and interquartile range 6.9 weeks). The questionnaires used for the interviews had close-ended questions with response choices for women were to choose from. The questionnaire can be accessed at: https://pma.ipums.org/pma/resources/questionnaires/mnh/PMAET_HQFQ_Panel_Cohort1_BL_Female_Questionnaire_v2.0_19May2021.pdf

### Measurements

The contact coverage was defined as the proportion of women who received four or more ANC visits during their most recent pregnancy. The input-adjusted effective ANC coverage was defined as the proportion of women receiving four or more ANC visits from healthcare facilities with skilled professionals, equipment, drugs, and supplies necessary for ANC service delivery, and emergency transport, as defined in Table 1. Data on the availability of the tracer items were collected by interviewing health care workers at the facilities, and directly observing some of the items using a predefined checklist. The facility readiness score was computed by taking the mean score of tracer items that should be available to provide ANC services at the healthcare facilities serving the included women [21]. In computing the facility readiness score, we included all health facilities that provide antenatal care for the included women within each EA. Input-adjusted ANC coverage was computed by multiplying the proportion of women who attended four or more ANC by facility readiness score.

**Table 1:** Services and tracer items used to measure facility readiness to provide antenatal care, PMA Ethiopia, 2019–2020

Availability of hemoglobin test
Availability of blood group test
Availability of blood glucose test
Availability of venereal disease research laboratory test
Availability of urine dipstick
Availability of fetoscope
Availability of blood pressure apparatus
Availability of weight scale
Availability of iron-folate supplementation
Availability of tetanus vaccination
Availability of private room for antenatal care
Access to emergency transport

Intervention-adjusted coverage was defined as the proportion of women receiving four or more ANC visits with both tetanus vaccination and iron-folate supplementation in the facility where the necessary inputs were available. These two were chosen as they are compulsory interventions of great importance for the woman and her newborn. Intervention-adjusted coverage was computed by multiplying the proportion who attended four or more ANC visits by facility readiness score and the proportion receiving both tetanus vaccination and iron-folate tablets.

The quality-adjusted coverage was defined as the proportion of women receiving four or more ANC visits and the two mentioned interventions per the recommended process quality at a facility where the necessary inputs were available (Table 2). The process quality score was calculated by taking the mean score of nine service components. Quality-adjusted coverage was computed by the proportion of women who received four or more ANC multiplied by the facility readiness score multiplied by intervention coverage multiplied by the process quality.

**Table 2:** Components included in antenatal care processes quality

Blood pressure measured
Weight taken
Blood sample taken
Urine sample taken
Provider discussed healthy diet
Provider discussed where to go for delivery
Provider discussed transport for delivery
Provider discussed dangers of bleeding before delivery
Provider discussed dangers of high blood pressure

### Effective ANC coverage

We took quality adjusted ANC coverage as a proxy measure of effective coverage, as we don’t have information on the other two components of the effective coverage cascade (client adherence and outcome adjusted coverage) as recommended by the “Effective coverage Think Tank Group” [11] in the PMA data.

### Data analysis

Background characteristics of women were summarized using descriptive summary measures. We used a STATA command svy to account for the clustered PMA data taking enumeration area as a primary sampling unit and households as a secondary sampling unit. The analysis was weighted by enumeration area and household to ensure the sample’s representativeness [19]. ANC coverage was stratified by region (Tigray, Afar, Amhara, Oromia, the Southern Nations, Nationalities, and Peoples region, and Addis Ababa) and urban or rural residence. We analyzed the proportion of women who completed four or more ANC visits to show the crude coverage of four or more ANC. We calculated the average facility readiness score by considering twelve essential items that should be available at health facilities to provide ANC services. To determine the input-adjusted ANC coverage, we multiplied the proportion of women who received four or more ANC visits by the average facility readiness score.

For intervention-adjusted ANC coverage, we multiplied the proportion of women who received four or more ANC visits by the average facility readiness score, and then by the proportion of women who received both iron-folate supplementation and tetanus toxoid vaccination. Lastly, we calculated the quality-adjusted ANC coverage by multiplying the proportion of women who received four or more ANC visits by the facility readiness score, the proportion who received iron-folate and tetanus toxoid, and the average process quality score based on nine quality components.

## Results

### Participation

**Fig. 1:**
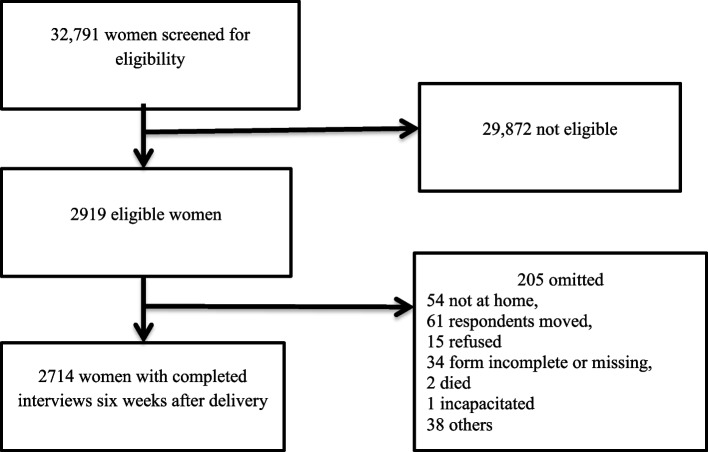
Study flow diagram, Performance Monitoring for Action Ethiopia, 2019–2020

Of the 2919 eligible women, 2714 completed the interview at around six weeks after delivery (93%) (Fig. 1). There were some differences between those who completed the study and dropped out of the study. The mean age for those women who completed the study was 27 years, while it was 25 years for those who dropped out of the study. Most women, who completed the study, were from rural areas (78%), while only 46% of the women who dropped from the study were from rural areas (Supplementary Table 1).

We conducted sensitivity analysis to see whether missing cases affected our results. For this purpose, we considered whether place of residence varied between those women who completed the repeat interview and missed ones. The number of women who completed the interview from rural residence was 1696, and the effective coverage for these rural women was 9%, while 1080 urban women completed the study, and the effective coverage was 21% for urban women. In comparison with the overall effective coverage of 12%, the sensitivity analysis effective coverage estimate was similar at 11.6%.

Most women were married (Table 3). Four out of ten had no formal education and a similar proportion had attended primary education. More than three-quarters were from rural areas. The median age of respondents was 27 years, (interquartile range of 8 years). Half of the mothers lived in households with five or more family members.

**Table 3:** Socio-demographic characteristics of study participants in Ethiopia, un-weighted distribution and weighted for enumeration area and household. Performance Monitoring for Action project, Ethiopia 2019–2020

**Characteristics of women**	**Unweighted** (*N* = 2714) *n* (%)	**Weighted** (*N* = 2714)%
**Age**		
15–18	170 (6)	8
19–34	2106 (78)	75
35–48	438(16)	17
**Parity**		
1	1140 (42)	38
2–4	1056 (39)	39
> = 5	518 (19)	23
**Marital status**		
Married	2641 (97)	98
Separated, divorced, widow, or single	73 (3)	2
**Education**		
No formal education	1049 (39)	42
Primary education	985 (36)	40
Secondary education	401 (15)	11
Technical and vocational education	109 (4)	4
Higher education	170 (6)	3
**Residence**		
Urban	1018 (38)	21
Rural	1696 (62)	79
**Region**		
Tigray	457 (17)	7
Afar	228 (8)	2
Amhara	465 (17)	20
Oromia	776 (25)	43
SNNP	638 (24)	23
Addis Ababa	250 (10)	4
**Household wealth quintiles**		
1 (lowest)	494 (18)	21
2	420 (15)	20
3	429 (16)	20
4	502 (19)	20
5 (highest)	869 (32)	19
**Family size**		
< 5	1426 (53)	49
5–8	1151 (42)	45
> 8	137 (5)	6

### Antenatal care coverage

The utilization of at least one ANC visit was 70% (95% CI, 64; 74), ranging from 14% in Afar to 85% in Tigray. Twenty-eight percent of women had their first ANC visit during the first trimester, 56% during the second, and 16% during the third trimester of pregnancy.

**Fig. 2:**
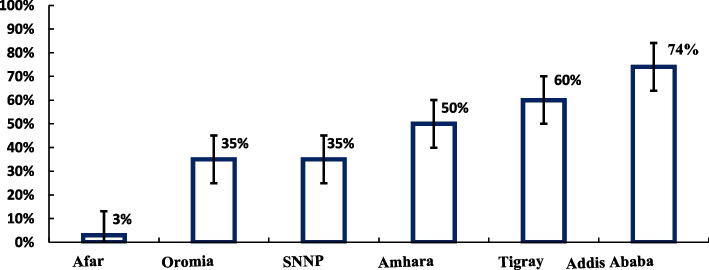
Proportion of women who completed four or more ANC visits across regions, PMA Ethiopia 2019–2020, *n* = 2714 (weighted and adjusted for clustering)

Forty percent of the participating women attended ANC four or more times (95% CI, 38; 43). This proportion varied across regions, ranging from 3% (95% CI, 2; 4) in Afar to 74% (95% CI, 59; 88) in Addis Ababa (Fig. 2). Overall, there were urban–rural differences with 65% (95% CI, 48; 86) for urban women and 33% (95% CI, 29; 39) for rural women.

Of the 605 health facilities assessed for service provision, we included 462 facilities in this study in computing facility readiness score (Table 4).

**Table 4:** Distribution of health facilities assessed for readiness to provide antenatal care in Ethiopia, by region, PMA 2019, *n* = 462

Region	Hospitals	Health Centers	Private and non-governmental facilities
Tigray	23 (38%)	27 (44%)	11 (18%)
Afar	6(33%)	12 (67%)	0
Amhara	33 (38%)	47 (53%)	8 (9%)
Oromia	39(33%)	49(42%)	30(25%)
SNNP	39 (35%)	42(38%)	30(27%)
Addis Ababa	6(9%)	22(33%)	38(58%)

SNNP: Southern Nations, and Nationalities, and Peoples’ region

### Input-adjusted ANC coverage

**Fig. 3:**
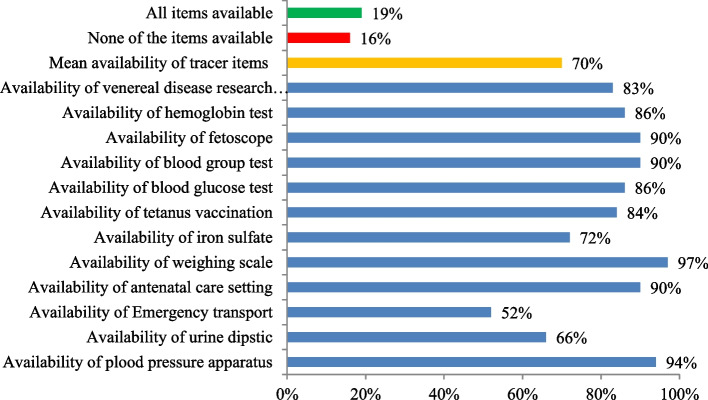
Proportion of items available at health care facilities to provide ANC, excluding health posts. PMA Ethiopia 2019–2020

Of the healthcare facilities providing services, 19% had all ANC services and tracer items, while 16% had none of them (Fig. 3). The mean overall availability of services and items necessary for ANC was 70%. Hence, the input-adjusted ANC coverage was 28% (40%*70%). The mean availability of items to provide ANC services was 94% for hospitals, 88% for health centers, and 57% for clinics. Clinics included private hospitals, higher and medium clinics and nongovernmental or faith-based health facilities. Stratifying by region, the mean facility readiness score was 76% for Tigray, 53% for Afar, 67% for Amhara, 70% for Oromia, 67% for SNNP, and 89% for Addis Ababa.

### Intervention-adjusted ANC coverage

Thirty percent of participating women had four or more ANC visits including tetanus vaccination and 35% had four or more ANC visits including iron-folate supplementation. If including both interventions, the coverage was 26%, and multiplied by the facility readiness score, the intervention-adjusted coverage was 18%. There were variations in intervention-adjusted coverage by region and place of residence (Table 5).

**Table 5:** Distribution of women who received four or more ANC along with tetanus toxoid and iron-folate by region and residence, Ethiopia, PMA 2019–2020, *n* = 2714

	Four or more ANC + TT and iron-folate
Region	
Tigray	30%
Afar	1.6%
Amhara	38%
Oromia	26%
SNNP	17%
Addis Ababa	48%
Residence	
Urban	40%
Rural	20%
Overall	26%

*TT* Tetanus toxoid vaccination

*SNNP* Southern Nations, Nationalities, and Peoples

### Quality-adjusted coverage

**Fig. 4:**
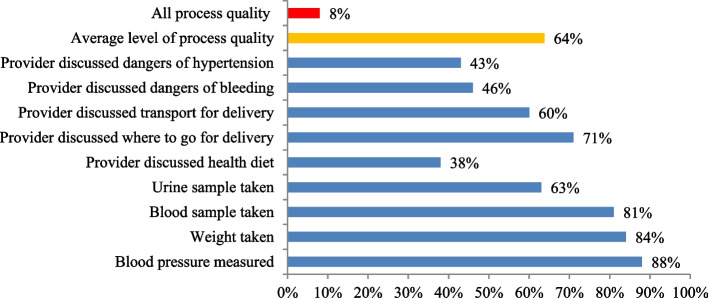
Proportion of essential ANC components received during ANC in Ethiopia, PMA 2019–2020

Among the essential ANC components, most women received screening for hypertension followed by weight monitoring (Fig. 4). Few women received counseling on healthy eating and discussions about the dangers of raised blood pressure during pregnancy. Nearly one in four did not receive any of the ANC components, while only 8% received four or more ANC visits along with all the ANC process quality components. The mean process quality score in this study was 64%. Among the women who attended four or more ANC visits, 45% received less than the mean process quality score. On average, the perceived quality score among urban women was 73% and it was 54% for rural women. The quality-adjusted ANC coverage (ANC4 *facility readiness score*intervention coverage*process quality score) was 9% for rural vs 21% for urban women.

### Effective ANC coverage

**Fig. 5:**
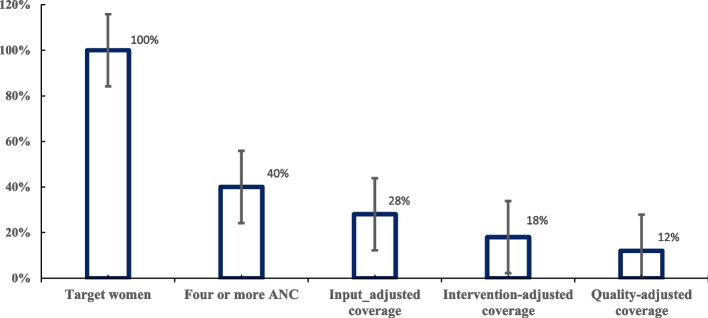
Effective ANC coverage in Ethiopia, PMA 2019–2020

The cascade of effective coverage is displayed in Fig. 5. After adjusting for facility readiness, intervention coverage and process quality scores, the quality-adjusted ANC coverage among the women included was 12%. Each bar in the figure below represents percentage of women who utilized the mentioned maternal health service.

## Discussion

Two-thirds of women had at least one antenatal care visit, of whom nearly half completed the recommended four or more visits. Considering the mean facility readiness score of 70%, on average, one in three women visited healthcare facilities ready to provide antenatal care. A quarter of women who completed four or more visits received both tetanus vaccination and iron-folate supplementation; hence, intervention-adjusted ANC coverage was 18%. The average level of process quality score for women who visited health facilities ready to provide ANC was 64%, therefore the quality-adjusted ANC coverage in this study was 12%. In other words, only 12% of women just one in every eight had received effective antenatal care.

Although most women initiated antenatal care visits, just 40% completed the recommended four or more visits. Our finding is lower than the estimate from a review of 32 sub-Saharan countries, where 55% completed four or more visits [22]. Half of the interviews were conducted during the COVID-19 pandemic which may have reduced the coverage of ANC visits. However, other studies showed a limited impact of the pandemic on ANC services uptake [23], and the pre-pandemic results in the EDHS 2019 report showed similar coverage levels. Distance from the health care facility, limited access to transportation, poor perceived ANC quality, and weak facility infrastructure could explain the low ANC uptake and completion of the recommended number of visits [24–26]. There was prominent geographic variation in the number of ANC visits with the highest in Addis Ababa (74%) and the lowest (3%) in Afar. These findings point to a need to design services that fit a mobile community.

Only one in three women received ANC at a facility where the necessary inputs were available. As expected, most public hospitals had the highest readiness score followed by public health centers and private and non-governmental health facilities. This finding aligns with a previous study showing low readiness to provide ANC at health centers, clinics, and health posts [27]. These findings imply that the health facilities close to the community were less prepared to provide ANC services.

Tetanus vaccination and iron-folate are two interventions that should be given to all pregnant women [21]. In this study, 30% of women received two doses of the vaccine, and 35% received iron-folate supplementation alongside four or more ANC. In line with our study, a review of 20 sub-Saharan countries showed that, although ANC service utilization has increased, only one in five women received tetanus vaccination and iron-folate supplementation, implying that poor ANC quality is a common problem [28].

Only 8% of the women received nine of the process quality components. This finding is lower than the previous report [18], which was from an earlier period and did not use the cascade approach when calculating effective coverage. Among the nine process quality components, the component with the least response was "discussion on healthy eating advice" followed by "discussion with the women about dangers of hypertension during pregnancy". A previous study, noted that giving more time to mothers during the first ANC visit was associated with the completion of the recommended number of ANC visits [29]. Effective communication with a pregnant woman will help a woman in building trust that could ensure utilization of the subsequent visits [30].

The overall effective ANC coverage of 12% is much lower than that reported in a review of 41 low- and middle-income countries [31], showing 60% effective coverage. However, the measurement approach used in the current study differed from the mentioned study in that they considered only the mean availability of ANC process quality components in computing effective ANC coverage, and in contrast we considered three components, the mean availability of items at health facilities, intervention coverage and the mean availability of ANC process quality components.

Ethiopia has made progress in increasing antenatal care coverage over the past three decades, with crude coverage nearly doubling [32]. Our analysis revealed a significant disparity between the crude antenatal care coverage and the quality-adjusted coverage with only one in ten women receiving high-quality care. This finding has important implications for policy and program development, highlighting the need for a greater focus on improving the quality of services and increasing access and coverage.

Effective health service coverage is a new approach that can be used as a proxy measure of the health system's performance to bring positive health outcomes. Quality of care plays a pivotal role in effective coverage, as it can impair health benefit gain and impacts the utilization of the subsequent maternal health services. The perceived low-quality of ANC services can discourage women from seeking the subsequent maternal health services. If a woman believes that the care provided at health facilities during ANC visits is suboptimal, she may choose home delivery, putting both her health and that of her baby at risk. Research indicates that the perceived poor quality of care at health facilities serves as a significant barrier to accessing maternal health services [33, 34]. This emphasizes the need to improve the quality of care during antenatal visits to encourage the utilization of subsequent maternal health services, ultimately leading to better maternal and newborn outcomes.

### Strengths and limitations

We measured effective ANC coverage using a community-based study representing 90% of the Ethiopian population. The study included women from the four agrarian regions (Tigray, Amhara, Oromia, and Southern Nations, Nationalities and Peoples), a pastoralist region (Afar), and from a city administration (Addis Ababa). Therefore, the findings of this study can be generalized to Ethiopia. The data were collected after meticulous training, including field practice and a pilot study that aimed to increase the study’s internal validity.

Our study had a short recall period of six weeks. Studies have demonstrated inconsistent validity of women's report of the maternal health services they received. Two studies conducted six months postpartum indicated that women's reports on services received during antenatal care were unreliable [35, 36] while one study, which utilized exit interviews, provided reliable information [37]. Based on this, our data collected within a relatively short period (six weeks) postpartum, is likely to have a minimal recall bias compared to a previous study that assessed effective ANC coverage for pregnancies in the five years before the survey from the nationally representative 2016 Ethiopian Demographic and Health Survey [14].

Among the seven percent of women who did not complete the study, urban women were over-represented. However, a sensitivity analysis showed no effect on the effective coverage estimate. As the study was based on self-reported interview data, social desirability bias might have affected women’s responses. This problem could potentially lead to an over-estimation of the coverage. However, ANC service is not a particularly sensitive issue, and given the thorough training of the data collectors, this is unlikely to have had a major impact on the result. In this study, 40% of mothers had no formal education, which could limit their understanding of some of the questions used to measure quality ANC. The questions that assessed the content of ANC received were not related to a specific ANC visit but were general. This limitation could have affected the process quality estimate.

We estimated input-adjusted coverage by linking the women's data on ANC use with the health facility that serves the area where the women lived. Some women may have bypassed the closest facility and attended services somewhere else. Previous studies showed that such linking resulted in acceptable estimates [20]. Although the two data sets were collected during the same period, skilled manpower, equipment, and supplies available at healthcare facilities might not have been the same when the women visited the facilities.

## Conclusions

The overall effective ANC coverage in Ethiopia was low, reflected in each component within the cascade. The overall low effective coverage was primarily a result of a considerable drop in contact coverage between the first ANC visit and the fourth ANC visit. Health facilities had on average a relatively fair readiness to provide ANC services. A relatively high proportion of women received tetanus vaccination and iron-folate supplementation. Women's reports of receiving services reflected suboptimal quality. Along with interventions increasing women’s awareness, improving quality of services is crucial to increase ANC uptake and completion of the recommended number of visits. The Ministry of Health, in collaboration with key decision-makers and stakeholders at all levels, must prioritize enhancing essential resources in healthcare facilities to ensure the provision of antenatal care. Strengthening existing initiatives, such as pre-service training, on-the-job training, mentorship, and supervision, is crucial to enhancing the quality of services offered. The low contact coverage for four or more ANC visits when the current guideline in Ethiopia recommends eight or more ANC visits indicates the need for implementation research that aim to enhance ANC uptake. We also encourage the Ministry of Health and other decision-makers to prioritize the use of effective coverage measurement using routine data when assessing service coverage.

### Supplementary Information

**Additional file 1: Table S1. **Characteristics of women who completed the study and missed from the study, PMA Ethiopia.

## Data Availability

The data for this manuscript were primarily collected by Addis Ababa University and Johns Hopkins Bloomberg School of Public Health to generate data on key indicators of reproductive, maternal, and newborn health. This dataset is publicly released. https://www.pmadata.org/countries/ethiopia.

## Abbreviations

ANC: Antenatal Care
EDHS: Ethiopian Demographic and Health Survey
HEW: Health Extension Workers
PMA: Performance Monitoring for Action

## References

[1] World Health Organization (WHO). WHO Recommendations on Antenatal Care for a Positive Pregnancy Experience: Summary. Geneva, Switzerland: WHO; 2018. Licence: CC BY-NC-SA 3.0 IGO 28079998

[2] Kuhnt J, Vollmer S (2017). Antenatal care services and its implications for vital and health outcomes of children: evidence from 193 surveys in 69 low-income and middle-income countries. BMJ Open.

[3] Chukwuma A, Wosu AC, Mbachu C, Weze K (2017). Quality of antenatal care predicts retention in skilled birth attendance: a multilevel analysis of 28 African countries. BMC Pregnancy Childbirth.

[4] Wondemagegn AT, Alebel A, Tesema C (2018). The effect of antenatal care follow-up on neonatal health outcomes: a systematic review and meta-analysis. Public Health Rev..

[5] Neogi SB, Sharma J, Negandhi P, Chauhan M, Reddy S, Sethy G (2018). Risk factors for stillbirths: how much can a responsive health system prevent?. BMC Pregnancy Childbirth.

[6] Benova L, Tunçalp Ö, Moran AC (2018). Not just a number: examining coverage and content of antenatal care in low-income and middle-income countries. BMJ Glob Health..

[7] Tesfaye N, Tariku R, Zenebe A, Dejene Z, Woldeyohannes F (2022). Cause and risk factors of early neonatal death in Ethiopia. PLoS One..

[8] Mahmood T, Rehman AU, Tserenpil G, Siddiqui F, Ahmed M, Siraj F, Kumar B (2019). The Association between Iron-deficiency Anemia and Adverse Pregnancy Outcomes: A Retrospective Report from Pakistan. Cureus.

[9] Jannati A, Sadeghi V, Imani A (2018). Effective coverage as a new approach to health system performance assessment: a scoping review. BMC Health Serv Res..

[10] Amouzou A, Leslie HH, Ram M, Fox M, Jiwani SS, Requejo J, Marchant T, Munos MK, Vaz LME, Weiss W, Hayashi C, Boerma T (2019). Advances in the measurement of coverage for RMNCH and nutrition: from contact to effective coverage. BMJ Glob Health.

[11] Andrew D, Marsh MM,Theresa Diaz, Jennifer Requejo, Debra Jackson, Doris Chou, Jenny A Cresswell, (2020). Effective coverage measurement in maternal, newborn, child, and adolescent health and nutrition: progress, future prospects, and implications for quality health systems. Lancet Glob Health..

[12] Tessema ZT, Teshale AB, Tesema GA, Tamirat KS (2021). Determinants of completing recommended antenatal care utilization in sub-Saharan from 2006 to 2018: evidence from 36 countries using Demographic and Health Surveys. BMC Pregnancy Childbirth.

[13] Arroyave L, Saad GE, Victora CG, Barros AJD (2021). Inequalities in antenatal care coverage and quality: an analysis from 63 low and middle-income countries using the ANCq content-qualified coverage indicator. Int J Equity Health.

[14] Institute Ethiopian Public Health, (EPHI) [Ethiopia] and ICF. Ethiopia Mini Demographic and Health Survey 2019: Key Indicators. Rockville, Maryland, USA: EPHI and ICF; 2019.

[15] Basha GW, Woya AA, Tekile AK (2020). Determinants of neonatal mortality in Ethiopia: an analysis of the 2016 Ethiopia Demographic and Health Survey. Afr Health Sci.

[16] Hailu GA, Weret ZS, Adasho ZA, Eshete BM (2022). Quality of antenatal care and associated factors in public health centers in Addis Ababa, Ethiopia, a cross-sectional study. PLoS ONE.

[17] Negash WD, Fetene SM, Shewarega ES (2022). Multilevel analysis of quality of antenatal care and associated factors among pregnant women in Ethiopia: a community based cross-sectional study. BMJ Open.

[18] Yakob B, Gage A, Nigatu TG, Hurlburt S, Hagos S, Dinsa G, Bowser D, Berman P, Kruk ME, Tekle E (2019). Low effective coverage of family planning and antenatal care services in Ethiopia. Int J Qual Health Care.

[19] Zimmerman L, Desta S, Yihdego M, Rogers A, Amogne A, Karp C, Wood SN, Creanga A, Ahmed S, Seme A, Shiferaw S (2020). Protocol for PMA-Ethiopia: A new data source for cross-sectional and longitudinal data of reproductive, maternal, and newborn health. Gates Open Res..

[20] Federal Minstry of Health, National Antenatal Care Guideline (2022). Ensuring Positive Pregnancy Experience! February 2022.

[21] Willey B, Waiswa P, Kajjo D, Munos M, Akuze J, Allen E, Marchant T (2018). Linking data sources for measurement of effective coverage in maternal and newborn health: what do we learn from individual- vs ecological-linking methods?. J Glob Health.

[22] Alem AZ, Shitu K, Alamneh TS (2022). Coverage and factors associated with completion of continuum of care for maternal health in sub-Saharan Africa: a multicountry analysis. BMC Pregnancy Childbirth.

[23] Amouzou A, Maïga A, Faye CM, Chakwera S, Melesse DY, Mutua MK (2022). a multicountry empirical assessment with a focus on maternal, newborn and child health services. BMJ Glob Health.

[24] Sisay G, Mulat T (2023). Antenatal Care Dropout and Associated Factors in Ethiopia: A Systematic Review and Meta-Analysis. Health Serv Res Manag Epidemiol.

[25] Alibhai KM, Ziegler BR, Meddings L, Batung E, Luginaah I (2022). Factors impacting antenatal care utilization: a systematic review of 37 fragile and conflict-affected situations. Confl Health.

[26] Singh R, Neogi SB, Hazra A, Irani L, Ruducha J, Ahmad D, Kumar S, Mann N, Mavalankar D (2019). Utilization of maternal health services and its determinants: a cross-sectional study among women in rural Uttar Pradesh, India. J Health Popul Nutr.

[27] Defar A, Getachew T, Taye G, Tadele T, Getnet M, Shumet T, Molla G, Gonfa G, Teklie H, Tadesse A, Bekele A (2020). Quality antenatal care services delivery at health facilities of Ethiopia, assessment of the structure/input of care setting. BMC Health Serv Res.

[28] Kanyangarara M, Munos MK, Walker N (2017). Quality of antenatal care service provision in health facilities across sub-Saharan Africa: Evidence from nationally representative health facility assessments. J Glob Health.

[29] Kumbeni MT, Apanga PA, Yeboah EO, Kolog JT, Awuni B (2021). The relationship between time spent during the first ANC contact, home visits and adherence to ANC contacts in Ghana. Glob Health Action.

[30] Lattof SR, Moran AC, Kidula N (2020). Implementation of the new WHO antenatal care model for a positive pregnancy experience: a monitoring framework. BMJ Global Health.

[31] Hodgins S, D'Agostino A (2014). The quality-coverage gap in antenatal care: toward better measurement of effective coverage. Glob Health Sci Pract.

[32] Tsegaye S, Yibeltal K, Zelealem H, Worku W, Demissie M, Worku A, Berhane Y (2022). The unfinished agenda and inequality gaps in antenatal care coverage in Ethiopia. BMC Pregnancy Childbirth.

[33] Sumankuuro J, Crockett J, Wang S (2018). Perceived barriers to maternal and newborn health services delivery: a qualitative study of health workers and community members in low and middle-income settings. BMJ Open.

[34] Toja E, Abebe A, Mekonen N, Baza D (2022). Why Home Delivery After Full Antenatal Care Follow-Up in Southern Ethiopia? An Exploratory-Descriptive Qualitative Study. Int J Womens Health.

[35] Bryce E, Katz J, Heidkamp R, Lama TP, Khatry SK, LeClerq S, Munos M (2022). Validation of maternal report of nutrition-related interventions and counselling during antenatal care in southern Nepal. Matern Child Nutr..

[36] Xie X, Munos MK, Lama TP, Bryce E, Khatry SK, LeClerq SC, Katz J (2023). Validation of maternal recall of number of antenatal care visits attended in rural Southern Nepal: a longitudinal cohort study. BMJ Open.

[37] McCarthy KJ, Blanc AK, Warren C (2020). validating women’s reports of antenatal and postnatal care received in Bangladesh. Cambodia and Kenya. BMJ Global Health.

