# Supplementary material for: Effective coverage of antenatal care services in Ethiopia: a population-based cross-sectional study
Source: BMC Pregnancy Childbirth. 2024 Apr 27;24:330. doi: 10.1186/s12884-024-06536-6 (PMC11055385; doi:10.1186/s12884-024-06536-6)
Supplement: Supplementary file 1 — Additional file 1: Table S1. Characteristics of women who completed the study and missed from the study, PMA Ethiopia. [file 12884_2024_6536_MOESM1_ESM.pdf]

Additional file

S Table 1: Characteristics of women who completed the study and missed from the study, PMA Ethiopia

Characteristics	Completed the interview n=2714	Missed N=205
Mean Age	27 years	25 years
Residence		
Urban	1020(38%)	104(54%)
Rural	1698(62%)	87(46%)
**Household wealth quintiles**		
1 (lowest)	494 (18)	14(7%)
2	420 (15)	26 (13%)
3	429 (16)	16 (12%)
4	502 (19)	49(24%)
5 (highest)	869 (32)	94(45%)
Educational status		
No formal education	1048 (38%)	55(26%)
Primary education	984(37%)	72(35%)
Secondary education	401(15%)	49(24%)
Technical and vocational	109(4%)	12(6%)
Tertiary education	170(6%)	19(9%)
